# Prevalence of antibiotic use for diarrhea among 1.3 million under-five years children: A multicounty retrospective analysis from 2006–2018

**DOI:** 10.1371/journal.pone.0289045

**Published:** 2023-08-15

**Authors:** Md. Shakil Ahmed, Suraiya Khanam, Md. Kamruzzaman, Mohammad Shahnewaz Morshed

**Affiliations:** 1 Department of Statistics, University of Rajshahi, Rajshahi, Bangladesh; 2 Department of Development Studies, Bangladesh University of Professionals (BUP), Dhaka, Bangladesh; 3 Institute of Bangladesh Studies, University of Rajshahi, Rajshahi, Bangladesh; 4 Institution of Statistical Research and Training (ISRT), University of Dhaka, Dhaka, Bangladesh; Nitte University, INDIA

## Abstract

**Background:**

This paper aims to estimate the prevalence of antibiotic use for diarrhea among under-five children (u5c) in low- and middle-income countries (LMICs) using data from 112 Demographic Health Surveys (DHS) conducted between 2006 and 2018. The focus is on understanding the extent of antibiotic usage for managing diarrhea, a condition characterized by frequent loose or watery bowel movements that can lead to severe dehydration.

**Methods and findings:**

A cross-sectional study design was employed in the DHS. The prevalence of antibiotic use for diarrhea among under-five children was estimated by analyzing DHS data from 2006 to 2018 and using the R statistical programming language. Out of a total of 12,69,944 children under five included in this study, 1,80,067 children had diarrhea and 19,502 children had bloody diarrhea. The overall prevalence of diarrhea estimated at ~14% (prevalence = 0.142; 95% CI = 0.141, 0.142). Among the children with diarrhea, 47,755 child received antibiotic treatment, resulting a prevalence of ~27% (prevalence = 0.27, 95% CI = 0.26, 0.27) globally. Central Asia had the highest prevalence of antibiotic use at ~55% (prevalence = (967/1748) = 0.55, 95% CI = 0.52, 0.59), followed by the Europe region with a prevalence of ~44% (prevalence = (5483/12502) = 0.44, 95% CI = 0.43, 0.45). In the South East and Central Asia region, DHS conducted between 2006 and 2018, showed the highest prevalence of antibiotic use in DHS 2007 (~44%), DHS 2012 (~49%), DHS 2016 (~40%) and DHS 2017 (~65%). The linear trend analysis showed an upward trend for using antibiotic of diarrhea in the South East and Central Asia region.

**Conclusions:**

The Central Asia region had the highest proportion of antibiotic use, with an estimated prevalence of ~55% (95% CI = 0.52, 0.59). The Europe region followed closely with a prevalence of ~44% (95% CI = 0.43, 0.45). The South East Asia region had the lowest prevalence of antibiotic use estimated at ~23% (95% CI = 0.22, 0.24), with a gradual increasing trend.

## Introduction

Diarrhea is defined as the occurrence of loose, liquid, or watery bowel movements three times or more per day [1]. Untreated persistent diarrhea can lead to severe dehydration. While vaccines can help prevent diarrhea-causing infections, antibiotic treatment is frequently administered alongside vaccines [2]. In low- and middle-income countries, diarrhea is a significant cause of antibiotic use in children. 13% of the population purchased antibiotics without a prescription, with 2% based on personal preference and 11% recommended by pharmacists [3]. The most prescribed drugs are Cotrimoxazole (51%), colistin sulfate (15.3%), norfloxacin (11%), and nalidixic acid (0.5%). Average number of antimicrobials used per inpatient case was higher than outpatients (1.15 vs 0.84, p < 0.001). Norfloxacin is commonly prescribed for childhood diarrhea [4]. In the last 20 years, the prevalence of antibiotic resistance has increased significantly [5]. It is a serious global health hazard [6]. The low- and middle-income countries face a greater threat of antibiotics depletion, higher prevalence of antibiotics use and antimicrobial resistance compared to high income countries [7]. The number of people accessing antimicrobial resistance is not only through formal health care but also through the increasing use of antibiotic treatments by both formal and informal health care providers [8, 9]. According to the worldwide Point Prevalence Survey, approximately one-third of patients admitted to hospital are prescribed antibiotic treatment [10]. In the Demographic Health Survey (DHS) and Multiple Indicator Cluster Survey (MICS), around 21% of antibiotics were administered orally (pills or syrups) and 3% through injections. The prevalence of antibiotic treatments for childhood diarrhea varied from 3% to 78% across 38 studies [11]. Modern healthcare is predominantly reliant on antibiotic treatment [12], but the improper and excessive use of antibiotics has led to emergence of resistant bacteria strains [13, 14]. In response to the overall issue of antimicrobial resistance (AMR) the World Health Organization (WHO) initiated various AMR-related activities, including the event of the Global Action Plan on Antimicrobial Resistance (GAP-AMR) during the 68th World Health Assembly in May 2015 [15]. Published literatures demonstrates a high proportion of inappropriate use of antimicrobials, highlighting the need to optimize the utilization of antimicrobial agents, which is one among the five key strategic objectives outlined within the GAP-AMR [15]. The main objective of this paper to estimate the prevalence of antibiotic use for diarrhea of under-five children (u5c) on the DHS multi-country survey from 2006–2018.

## Methods

### Data sources

In this study, data from 112 Demographic Health Survey (DHS) datasets was utilized. According to the DHS protocol, detailed questioned were asked to mothers during household surveys about the management of diarrhea episodes. In the DHS, the data collected specifically focused on u5c who had experienced 2-weeks of diarrhea episodes prior to the survey date. The datasets used in this study consist of a total of 12,69,944 records for u5c obtained from 112 DHS programmed surveys conducted between 2006 and 2018 (https://dhsprogram.com/Data/). The DHS employs a cross-sectional sampling strategy and methodology, which are extensively described on the DHS website and published reports.

### Statistical analysis

#### Outcome variable or dependent variable

The outcome variable in this study is antibiotic use for diarrheal disease among the under-five children in DHS survey regions from 2006 to 2018.

#### Antibiotic treatment response category

Given antibiotic pills or syrups:

0: No

1: Yes; oth pill, syrup

8: Don’t know

9: Missing

#### Independent variable or exposure variables

Mother education, wealth index, age in months, All the statistical analysis were performed using the open-source software R statistical programming language (https://www.r-project.org/). Descriptive analysis was conducted to determine the prevalence of diarrhea and antibiotic use. For each estimate, a 95% confidence interval was calculated to provide a measure of the precision of the estimate. A Linear trend model was used to analyze the antibiotic use trend from 2006 to 2018.

## Results

### Overall characteristics

Out of the total 1,269,944 children under the age of five included in this study, 180,067 had diarrhea and 19,502 had bloody diarrhea. Among all types of diarrheas, 47,755 children received antibiotic treatment. The overall prevalence of diarrhea was estimated to be ~14% (prevalence = 0.142; 95% CI = 0.141, 0.142). The prevalence of bloody diarrhea was ~2% (prevalence = 0.015; 95% CI = 0.015, 0.016). The prevalence of antibiotic treatment for diarrhea among children under the age of five was ~27% (prevalence = 0.27; 95% CI = 0.26, 0.27) in the DHS regions worldwide.

### Socio-economic and demographic characteristics

**Fig 1:**
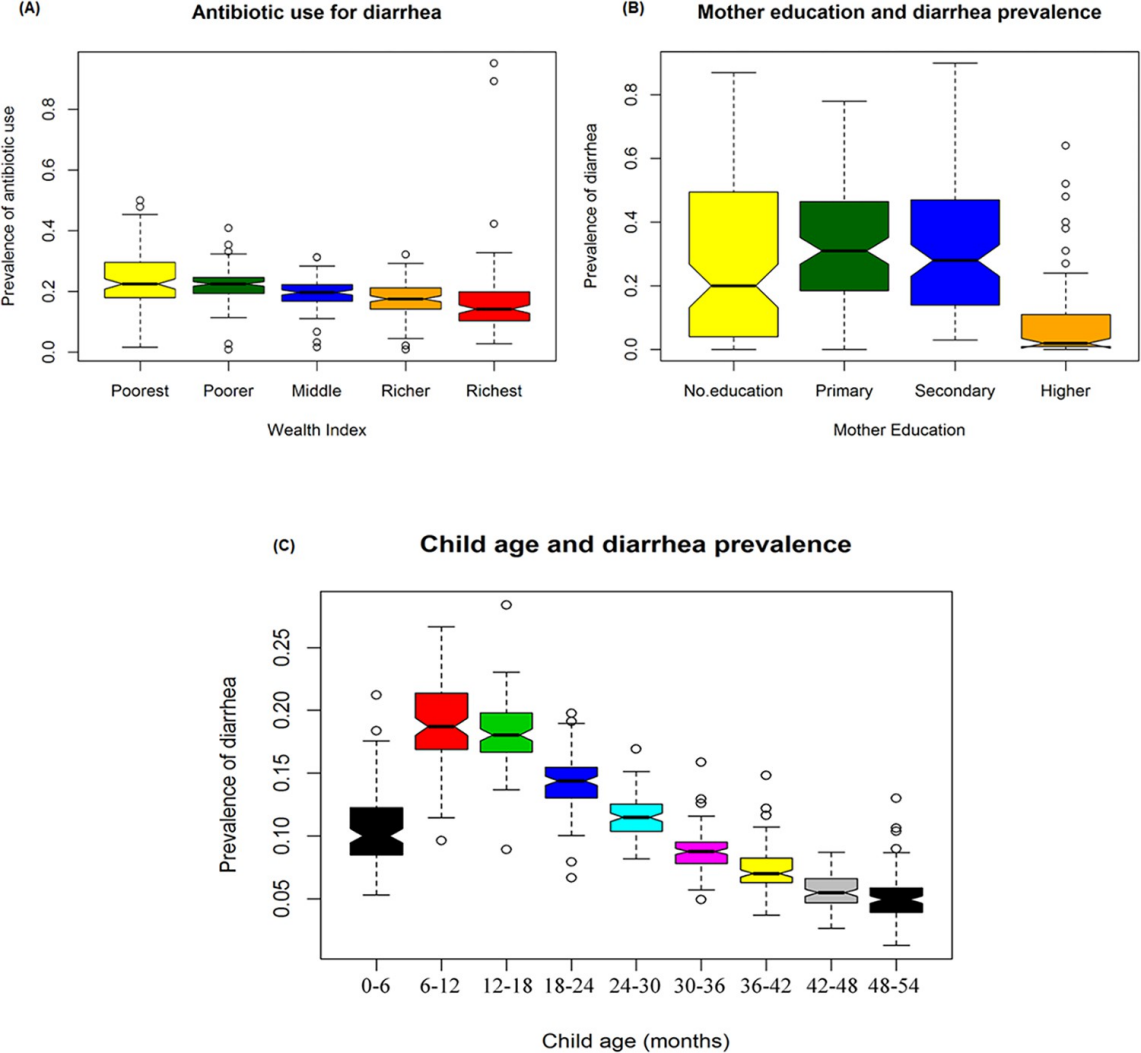
Box plot for (A) prevalence of antibiotic use by wealth index, (B) prevalence of diarrhea by mother education, (C) prevalence of diarrhea at different age groups of several DHS survey regions.

The wealth index is a significant indicator of socio-economic status. The median prevalence (robust measures of central tendency with 50% outliers tolerate) of antibiotic use for diarrhea was almost equal among the poorer (~22%) and poorest (~22%) group of children. In contrast, the prevalence of antibiotic use for diarrhea was ~14% among the richest individuals. Therefore, the box plot (**Fig 1(A)**) indicates a decreasing trend in prevalence of antibiotic use from poorest to the richest group (**Table 1)**.

**Table 1:** Wealth index descriptive statistics of prevalence of antibiotic use.

Poorest	Poorer	Middle	Richer	Richest
Min.: 0.01587	Min.: 0.007937	Min.: 0.01587	Min.: 0.007937	Min.: 0.02732
1st Qu.:0.17941	1st Qu.:0.193364	1st Qu.:0.16914	1st Qu.:0.143732	1st Qu.:0.10356
Median: 0.22420	Median: 0.224445	Median: 0.19663	Median: 0.175391	Median: 0.14186
Mean: 0.23906	Mean: 0.220693	Mean: 0.19550	Mean: 0.174874	Mean: 0.16987
3rd Qu.:0.29513	3rd Qu.:0.245749	3rd Qu.:0.22208	3rd Qu.:0.211032	3rd Qu.:0.19841
Max.: 0.50000	Max.: 0.409091	Max.: 0.31281	Max.: 0.321267	Max.: 0.95238

Mother’s education plays a crucial role in under five diarrhea prevalence. The prevalence of diarrhea was higher, at ~31% (median), among children whose mothers had primary education and ~28% (median) among children whose mothers had secondary education. The prevalence of diarrhea was comparatively lower ~20% (median) among children whose mother had no education. However, the prevalence of diarrhea remarkably lowers ~2% among the higher education mother (**Fig 1(B)** and **Table 2**).

**Table 2:** Mother education descriptive statistics for prevalence of diarrhea.

No. education	Primary	Secondary	Higher
Min.: 0.0000	Min.: 0.0000	Min.: 0.0300	Min.: 0.00000
1st Qu.:0.0400	1st Qu.:0.1850	1st Qu.:0.1400	1st Qu.:0.01000
Median: 0.2000	Median: 0.3100	Median: 0.2800	Median: 0.02000
Mean: 0.2863	Mean: 0.3369	Mean: 0.3114	Mean: 0.07856
3rd Qu.:0.4950	3rd Qu.:0.4650	3rd Qu.:0.4700	3rd Qu.:0.11000
Max.: 0.8700	Max.: 0.7800	Max.: 0.9000	Max.: 0.64000

The prevalence of diarrhea was higher among the age group of 6–12 months and 12–18 months, with medians of ~19% and ~18% respectively. In contrast, the prevalence of diarrhea was very low, at ~5% (median), among the age groups of 42–48 months and 48–54 months (Table 3). The box plot (Fig 1(C)) illustrates a decreasing trend in the prevalence of diarrhea from the age group of 6–12 months to 48–54 months.

**Table 3:** Descriptive statistics for prevalence of diarrhea for all age groups.

Descriptive statistics	0–6	6–12	12–18	18–24	24–30	30–36	36–42	42–48	48–54
Minimum	0.05310	0.0963	0.08943	0.06667	0.08182	0.04938	0.03704	0.02654	0.01292
Q1	0.08503	0.1689	0.16695	0.13043	0.10397	0.07830	0.06312	0.04713	0.03928
Median	0.10022	0.1871	0.18042	0.14388	0.11489	0.08775	0.07024	0.05502	0.04951
Mean	0.10442	0.1886	0.18214	0.14229	0.11449	0.08743	0.07361	0.05651	0.05053
Q3	0.12256	0.2137	0.19813	0.15468	0.12533	0.09516	0.08257	0.06614	0.05872
Maximum	0.21231	0.2665	0.28395	0.19778	0.16928	0.15873	0.14815	0.08705	0.13008

### Global hotspot of antibiotic use for under-five children diarrhea

**Fig 2:**
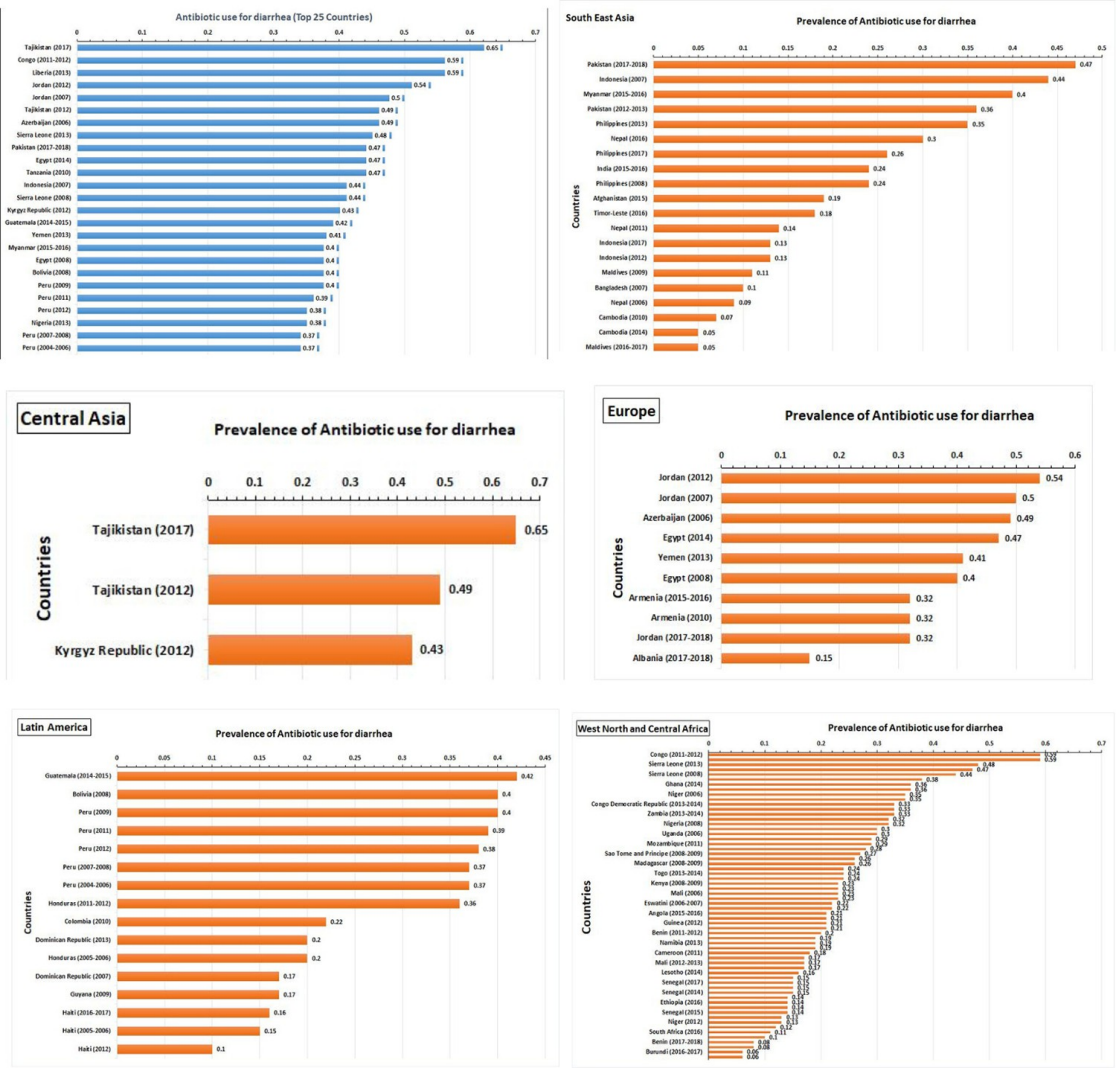
Prevalence of antibiotic use for under-five children diarrhea among the DHS countries.

Among all DHS regions, 25 countries have been identified with the highest prevalence of antibiotic use for the treatment of childhood diarrhea, ranging from 37% to 65% (Fig 2). In the South East Asia DHS region, the top three hotspot countries with the highest prevalence of antibiotic use among children under the age of five were Pakistan (2017–2018) with 47%, Indonesia (2007) with 44%, and Myanmar (2015–2016) with 40%. In Central Asia, Tajikistan (2017) had the highest prevalence of antibiotic use at 65%. In the Europe region, Jordan (2012 and 2007) had a prevalence of antibiotic use for diarrhea treatment exceeding 50%. In the Latin America region, Guatemala (2014–2015), Bolivia (2008), and Peru (2009) had prevalence rates of antibiotic use for diarrhea treatment exceeding 40%. In the Africa region, Congo (2011–2012) and Sierra Leone (2013 and 2008) had approximately 59% of people using antibiotics for the treatment of under-five children with diarrhea (Fig 2).

### Prevalence of antibiotic use for under five children diarrhea

In the central Asia region, a total of 15,089 children under the age of five were included in the survey. Among them, 1,748 Children had diarrhea and 967 Children had received antibiotic treatment for diarrhea. The prevalence of antibiotic use for diarrhea in Central Asia region was ~55% (prevalence = (967/1748) = 0.55, 95% CI = 0.52, 0.59). In the Europe DHS region, the prevalence of antibiotic for diarrhea was ~44% (prevalence = (5483/12502) = 0.44, 95% CI = 0.43, 0.45). The lowest prevalence of antibiotic use for children under the age of five with diarrhea was ~23% (prevalence = (11918/51328) = 0.23, 95% CI = 0.22, 0.24) in the South East Asia DHS region. In the Latin America, and West North and Central Africa DHS region, the prevalence of antibiotic use for diarrhea were ~30% (prevalence = (7887/26396) = 0.30, 95% CI = 0.29, 0.31) and ~24% (prevalence = (21500/88093) = 0.24, 95% CI = 0.23, 0.24) respectively (**Table 4**).

**Table 4:** Prevalence of antibiotic treatment for diarrhea of under-five child (u5c) among 112 DHS national survey 2006–2018.

DHS Survey Countries	Total No. of u5c	No. of u5c diarrhea	No. of u5c antibiotic use for diarrhea	Antibiotic use for diarrhea
**South East Asia**	**N**	**n**	**n**	**Prevalence (95% CI)**
Afghanistan (2015)	30951	7990	1522	0.19 (0.17, 0.21)
Bangladesh (2007)	5789	560	56	0.1 (0.02, 0.18)
Cambodia (2014)	6970	855	45	0.05 (-0.01, 0.12)
Cambodia (2010)	7820	1135	83	0.07 (0.02, 0.13)
India (2015–2016)	247743	22500	5464	0.24 (0.23, 0.25)
Indonesia (2017)	17263	2440	306	0.13 (0.09, 0.16)
Indonesia (2012)	17323	2505	321	0.13 (0.09, 0.16)
Indonesia (2007)	17891	2536	1104	0.44 (0.41, 0.46)
Maldives (2016–2017)	3055	126	6	0.05 (-0.18, 0.27)
Maldives (2009)	3759	188	20	0.11 (-0.04, 0.25)
Myanmar (2015–2016)	4596	550	219	0.4 (0.33, 0.46)
Nepal (2016)	4861	336	102	0.3 (0.21, 0.39)
Nepal (2011)	5054	679	93	0.14 (0.07, 0.21)
Nepal (2006)	5457	659	61	0.09 (0.02, 0.17)
Pakistan (2017–2018)	11985	2107	985	0.47 (0.44, 0.5)
Pakistan (2012–2013)	10935	2298	829	0.36 (0.33, 0.39)
Philippines (2017)	10297	652	171	0.26 (0.2, 0.33)
Philippines (2013)	7012	551	194	0.35 (0.28, 0.42)
Philippines (2008)	6382	571	139	0.24 (0.17, 0.32)
Timor-Leste (2016)	6950	700	124	0.18 (0.11, 0.25)
Timor-Leste (2009–2010)	9294	1390	74	0.05 (0, 0.11)
**Total**	**441387**	**51328**	**11918**	**0.23 (0.22, 0.24)**
**Central Asia**	** **	** **		
Kyrgyz Republic (2012)	4236	223	95	0.43 (0.33, 0.53)
Tajikistan (2017)	6019	802	519	0.65 (0.61, 0.69)
Tajikistan (2012)	4834	723	353	0.49 (0.44, 0.54)
**Total**	**15089**	**1748**	**967**	**0.55 (0.52, 0.59)**
**Europe**	** **	** **		
Albania (2017–2018)	2755	149	22	0.15 (-0.01, 0.3)
Armenia (2015–2016)	1709	68	22	0.32 (0.12, 0.53)
Armenia (2010)	1450	128	41	0.32 (0.17, 0.47)
Azerbaijan (2006)	2196	231	113	0.49 (0.4, 0.58)
Egypt (2014)	15465	2010	949	0.47 (0.44, 0.5)
Egypt (2008)	10595	979	390	0.4 (0.35, 0.45)
Jordan (2017–2018)	10475	968	312	0.32 (0.27, 0.37)
Jordan (2012)	10128	1540	830	0.54 (0.5, 0.57)
Jordan (2007)	10237	1659	836	0.5 (0.47, 0.54)
Yemen (2013)	15326	4770	1968	0.41 (0.39, 0.43)
**Total**	**80336**	**12502**	**5483**	**0.44 (0.43, 0.45)**
**Latin America**	** **	** **		
Bolivia (2008)	8193	2055	825	0.4 (0.37, 0.43)
Colombia (2010)	17443	2495	549	0.22 (0.19, 0.25)
Dominican Republic (2013)	3605	637	125	0.2 (0.13, 0.27)
Dominican Republic (2007)	10796	1773	294	0.17 (0.12, 0.21)
Guatemala (2014–2015)	12068	2239	946	0.42 (0.39, 0.45)
Guyana (2009)	2105	213	36	0.17 (0.04, 0.3)
Haiti (2016–2017)	6120	1235	202	0.16 (0.11, 0.21)
Haiti (2012)	6744	1415	141	0.1 (0.05, 0.15)
Haiti (2005–2006)	5596	1217	182	0.15 (0.1, 0.2)
Honduras (2011–2012)	10592	1919	684	0.36 (0.32, 0.39)
Honduras (2005–2006)	10506	1797	360	0.2 (0.16, 0.24)
Peru (2012)	9445	1254	479	0.38 (0.34, 0.43)
Peru (2011)	8950	1312	518	0.39 (0.35, 0.44)
Peru (2009)	10041	1475	586	0.4 (0.36, 0.44)
Peru (2007–2008)	16730	2680	980	0.37 (0.34, 0.4)
Peru (2004–2006)	16730	2680	980	0.37 (0.34, 0.4)
**Total**	**155664**	**26396**	**7887**	**0.30 (0.29, 0.31)**
**West North and Central Africa**	** **	** **		
Angola (2015–2016)	13619	1891	406	0.21 (0.17, 0.25)
Benin (2017–2018)	12651	1342	101	0.08 (0.02, 0.13)
Benin (2011–2012)	12679	816	163	0.2 (0.14, 0.26)
Burkina Faso (2010)	13716	2031	576	0.28 (0.25, 0.32)
Burundi (2016–2017)	12472	2664	151	0.06 (0.02, 0.09)
Burundi (2010)	7231	1787	384	0.21 (0.17, 0.26)
Cameroon (2011)	10713	2078	372	0.18 (0.14, 0.22)
Chad (2014–2015)	16837	3292	474	0.14 (0.11, 0.18)
Comoros (2012)	3022	480	91	0.19 (0.11, 0.27)
Congo (2011–2012)	8857	1531	908	0.59 (0.56, 0.63)
Congo (2005)	4435	627	203	0.32 (0.26, 0.39)
Congo Democratic Republic (2013–2014)	17188	2818	939	0.33 (0.3, 0.36)
Congo Democratic Republic (2007)	7987	1287	336	0.26 (0.21, 0.31)
Cote d’Ivoire (2011–2012)	7052	1276	163	0.13 (0.08, 0.18)
Eswatini (2006–2007)	2537	347	75	0.22 (0.12, 0.31)
Ethiopia (2016)	10006	1090	150	0.14 (0.08, 0.19)
Ethiopia (2011)	10808	1620	251	0.15 (0.11, 0.2)
Gabon (2012)	5747	981	299	0.3 (0.25, 0.36)
Gambia (2013)	7788	1340	325	0.24 (0.2, 0.29)
Ghana (2014)	5593	671	243	0.36 (0.3, 0.42)
Ghana (2008)	2794	553	201	0.36 (0.3, 0.43)
Guinea (2012)	6396	1071	226	0.21 (0.16, 0.26)
Kenya (2014)	20069	2953	505	0.17 (0.14, 0.2)
Kenya (2008–2009)	5706	946	217	0.23 (0.17, 0.29)
Lesotho (2014)	2915	328	52	0.16 (0.06, 0.26)
Liberia (2013)	7058	1675	989	0.59 (0.56, 0.62)
Liberia (2007)	5305	1072	148	0.14 (0.08, 0.19)
Madagascar (2008–2009)	11750	1006	260	0.26 (0.2, 0.31)
Malawi (2015–2016)	16462	3402	979	0.29 (0.26, 0.32)
Malawi (2010)	18360	3105	717	0.23 (0.2, 0.26)
Mali (2012–2013)	9582	844	140	0.17 (0.1, 0.23)
Mali (2006)	12388	1450	335	0.23 (0.19, 0.28)
Mozambique (2011)	10291	1071	313	0.29 (0.24, 0.34)
Namibia (2013)	4805	810	156	0.19 (0.13, 0.25)
Namibia (2006–2007)	4841	576	112	0.19 (0.12, 0.27)
Niger (2012)	11602	1591	199	0.13 (0.08, 0.17)
Niger (2006)	8209	1669	590	0.35 (0.31, 0.39)
Nigeria (2013)	28596	2968	1121	0.38 (0.35, 0.41)
Nigeria (2008)	25273	2645	849	0.32 (0.29, 0.35)
Rwanda (2014–2015)	7556	905	106	0.12 (0.06, 0.18)
Rwanda (2010)	8484	1109	116	0.1 (0.05, 0.16)
Sao Tome and Principe (2008–2009)	1851	230	61	0.27 (0.15, 0.38)
Senegal (2017)	11605	2212	336	0.15 (0.11, 0.19)
Senegal (2016)	6417	1062	164	0.15 (0.1, 0.21)
Senegal (2015)	6602	1359	195	0.14 (0.09, 0.19)
Senegal (2014)	6526	1272	186	0.15 (0.1, 0.2)
Senegal (2012–2013)	6540	972	201	0.21 (0.15, 0.26)
Senegal (2010–2011)	11633	2196	494	0.22 (0.19, 0.26)
Sierra Leone (2013)	10618	1214	581	0.48 (0.44, 0.52)
Sierra Leone (2008)	5043	590	258	0.44 (0.38, 0.5)
South Africa (2016)	3413	350	37	0.11 (0, 0.21)
Tanzania (2015–2016)	9707	1125	369	0.33 (0.28, 0.38)
Tanzania (2010)	7526	1015	478	0.47 (0.43, 0.52)
Togo (2013–2014)	6530	1042	251	0.24 (0.19, 0.29)
Uganda (2016)	14710	2923	666	0.23 (0.2, 0.26)
Uganda (2011)	7355	1684	589	0.35 (0.31, 0.39)
Uganda (2006)	7593	1956	579	0.3 (0.26, 0.33)
Zambia (2013–2014)	12698	2045	670	0.33 (0.29, 0.36)
Zambia (2007)	5844	909	221	0.24 (0.19, 0.3)
Zimbabwe (2015)	5807	931	72	0.08 (0.01, 0.14)
Zimbabwe (2010–2011)	5203	674	116	0.17 (0.1, 0.24)
Zimbabwe (2005–2006)	4867	614	35	0.06 (-0.02, 0.14)
**Total**	**577468**	**88093**	**21500**	**0.24 (0.23, 0.24)**
**Grand Total**	**1269944**	**180067**	**47755**	**0.27 (0.26, 0.27)**

#### South East Asia

**Fig 3:**
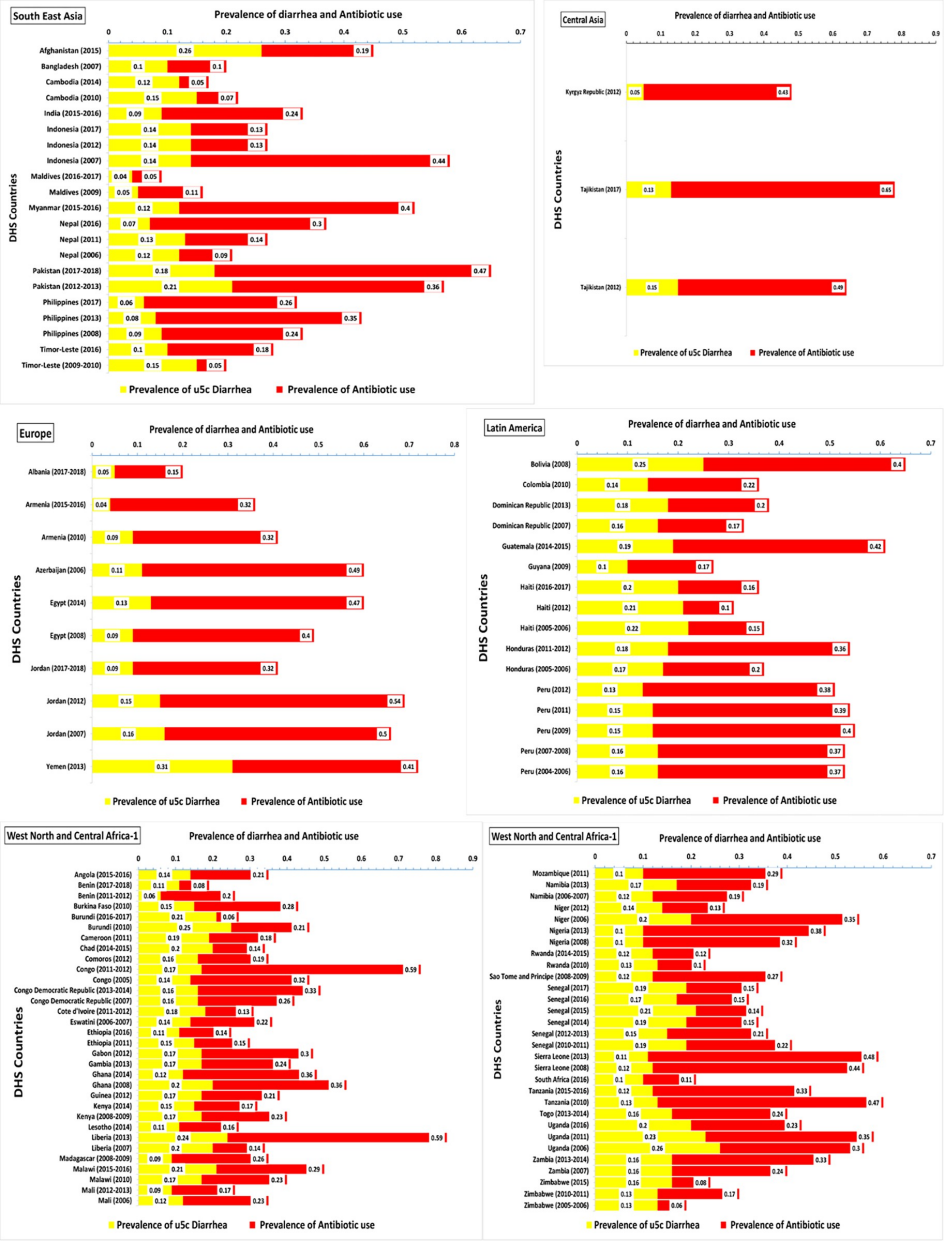
Prevalence of diarrhea and prevalence of antibiotic use of under five years children among 112 DH survey regions.

In the South East Asia region, Pakistan (2017–2018) and Indonesia (2007) had the highest prevalence of antibiotic use for diarrhea. The estimated prevalence was ~47% (prevalence = 0.47, 95% CI = 0.44, 0.50) in Pakistan and ~44% (prevalence = 0.44, 95% CI = 0.41, 0.46) in Indonesia. The lowest prevalence of antibiotic use for diarrhea was ~5% (prevalence = 0.05, 95% CI = -0.18, 0.27) in Maldives (2016–2017) and ~5% (prevalence = 0.05, 95% CI = 0.0, 0.11) in Timor-Leste (2009–2010) (**Table 4** & **Fig 3**).

#### Central Asia

In Central Asia region, the prevalence of antibiotic use for diarrhea was ~40%. Tajikistan (2017) had the highest prevalence ~65% (prevalence = 0.65, 95% CI = 0.61, 0.69) of antibiotic use for Child diarrhea (**Table 4** & **Fig 3**).

#### Europe

The highest prevalence of antibiotic use for diarrhea was observed ~54% (prevalence = 0.54, 95% CI = 0.50, 0.57) in Jordan (2012) and ~49% (prevalence = 0.49, 95% CI = 0.40, 0.58) in Azerbaijan (2006). The lowest prevalence of antibiotic use for diarrhea was ~15% (prevalence = 0.15, 95% CI = -0.01, 0.30) in Albania (2017–2018) (**Table 4** & **Fig 3**).

#### Latin America

In the Latin America DHS region, Guatemala (2014–2015) had the highest prevalence of antibiotic use for diarrhea, estimated at ~42% (prevalence = 0.42, 95% CI = 0.39, 0.45). Other countries with high prevalence rates include Bolivia (2008) with an estimated prevalence of ~40% (prevalence = 0.40, 95% CI = 0.37, 0.43), Peru (2009) with ~39% (prevalence = 0.39, 95% CI = 0.35, 0.44), Peru (2011) with ~38% (prevalence = 0.38, 95% CI = 0.34, 0.43) and Peru (2012) with ~40% (prevalence = 0.40, 95% CI = 0.36, 0.44). Haiti (2012) had the lowest prevalence of antibiotic use for diarrhea estimated at ~10% (prevalence = 0.10, 95% CI = 0.05, 0.15) (**Table 4** & **Fig 3**).

#### West North and Central Africa

In the West North and Central Africa region, Congo (2011–2012) had the highest prevalence of antibiotic use for diarrhea with an estimated prevalence of ~59% (prevalence = 0.59, 95% CI = 0.56, 0.63), followed by Liberia (2013) with a similar prevalence of ~59% (prevalence = 0.59, 95% CI = 0.56, 0.62). Burundi (2016–2017) with a prevalence of ~6% (prevalence = 0.06, 95% CI = 0.02, 0.09) and Zimbabwe (2005–2006) with prevalence of ~6% (prevalence = 0.06, 95% CI = -0.02, 0.14) had the lowest prevalence of antibiotic use for diarrhea. Sierra Leone (2008), Sierra Leone (2013) and Tanzania (2010) had higher prevalence of antibiotic use for diarrhea with prevalence of ~48% (prevalence = 0.48, 95% CI = 0.44, 0.52), ~44% (prevalence = 0.44, 95% CI = 0.38, 0.50) and ~47% (prevalence = 0.47, 95% CI = 0.43, 0.52) respectively (**Table 4 & Fig 3**).

### The trend of antibiotic use for diarrhea

#### South East & Central Asia

**Fig 4:**
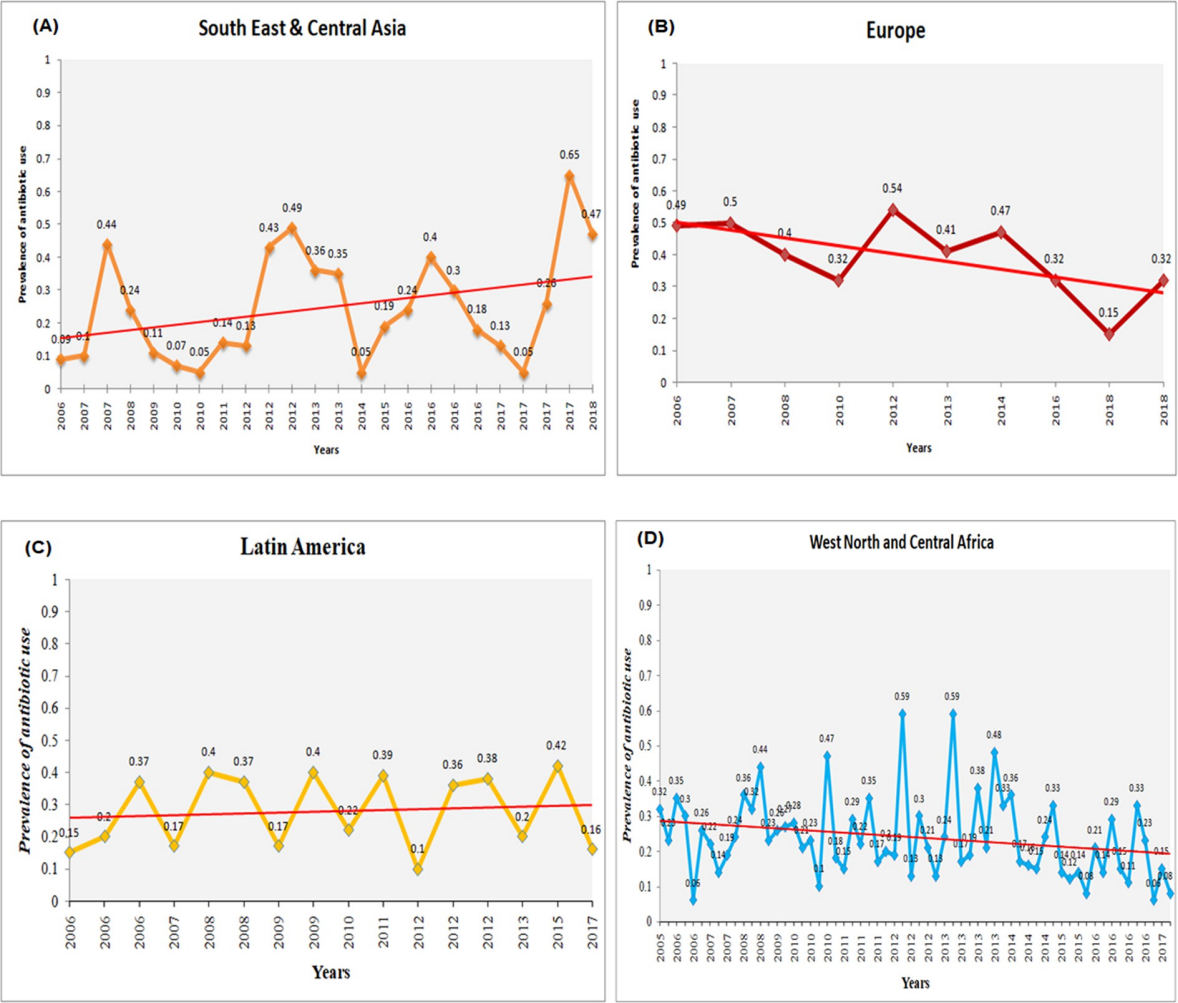
Linear trend analysis of antibiotic use for diarrhea among (A) Southeast and Central Asia, (B) Europe, (C) Latin America, and (D) West North and Central Africa.

The prevalence of antibiotic use for under five diarrhea in the South East & Central Asia region showed an increasing trend from 2006 to 2018. The highest rates observed in DHS 2017 (~65%), DHS 2016 (~40%), DHS 2012 (~49%) and DHS 2007 (~44%). The linear trend analysis showed an upward trend in the use of antibiotics for diarrhea (**Fig 4**(**A**)).

#### Europe

The prevalence of antibiotic use for under five diarrhea in the Europe region showed a decreasing trend between 2007 and 2018. The highest prevalence estimated in DHS 2012 (~54%) followed by DHS 2007 (~50%), DHS 2014 (~47%) and DHS 2018 (~32%). The linear trend analysis confirmed downward trend in antibiotic use for diarrhea in the Europe DH survey region (**Fig 4**(**B**)).

#### Latin America

The Latin America region showed a decreasing trend in the prevalence of antibiotic use between 2008 and 2018. The highest prevalence rates were estimated in DHS 2008 (~40%) followed by DHS 2009 (~40%), DHS 2006 (~37%) and DHS 2015 (~32%). The linear trend analysis showed a declining trend in the use of antibiotics for diarrhea in the region (**Fig 4**(**C**)).

#### West North and Central Africa

The prevalence of antibiotic use for under-five children with diarrhea in the West North and Central Africa region varied across the DHS conducted from 2006 to 2018. The highest estimated prevalence was ~59% in DHS 2012, while the lowest estimated at ~8% in DHS 2018. DHS 2008 (~44%), DHS 2010 (~47%) and DHS 2013 (~48%) had relatively higher prevalence rates. The linear trend analysis revealed a declining trend of antibiotic use for diarrhea in this DHS region (**Fig 4**(**D**)).

## Discussions

Antibiotic use for diarrhea is prevalent among under-five children in low and middle-income countries. The overall prevalence of antibiotic treatment for diarrhea among under-five children worldwide estimated at ~27% (prevalence = 0.27, 95% CI = 0.26, 0.27). Among specific regions, Central Asia had the highest prevalence of antibiotic use estimated at ~55% (prevalence = (967/1748) = 0.55, 95% CI = 0.52, 0.59), followed by the Europe region with a prevalence of ~44% (prevalence = (5483/12502) = 0.44, 95% CI = 0.43, 0.45). The lowest prevalence of antibiotic use estimated at ~23% (prevalence = (11918/51328) = 0.23, 95% CI = 0.22, 0.24) in the South East Asia region. Conversely, the linear trend analysis indicates an upward trend in the use of antibiotics for diarrhea in the South East and Central Asia region. The West North and Central Africa DHS region showed a decreasing trend in antibiotic use for under-five with diarrhea.

In summary, Central Asia and Europe have the highest proportions of antibiotic use for diarrhea among under-five children, with Central Asia showing the highest prevalence. Conversely, the trend analysis reveals an increasing trend in South East and Central Asia and decreasing trends in Europe, Latin America, and West North and Central Africa regions.
